# The dysbiosis of gut microbiota and dysregulation of metabolites in IgA nephropathy and membranous nephropathy

**DOI:** 10.3389/fmed.2025.1618947

**Published:** 2025-07-16

**Authors:** Lei Zhang, Lan Hu, Li Tan, Zhenjie Zhang, Mengying Chen, Wenbo Gan, Li Chen, Yan Zou, Shi Wang, Yu Pang, Zhenxin Fan, Junjie Liu

**Affiliations:** ^1^Guang’an People’s Hospital, Guan’an, China; ^2^Department of Emergency Medicine, West China Hospital, West China School of Nursing, Sichuan University, Chengdu, China; ^3^Institute of Disaster Medicine, Sichuan University, Chengdu, China; ^4^Nursing Key Laboratory of Sichuan Province, Chengdu, China; ^5^Key Laboratory of Bioresources and Ecoenvironment (Ministry of Education), College of Life Sciences, Sichuan University, Chengdu, China

**Keywords:** IgA nephropathy, membranous nephropathy, gut microbiota, metabolomics, biomarker

## Abstract

**Introduction:**

Immunoglobulin A nephropathy (IgAN) and membranous nephropathy (MN) are among the most common forms of primary glomerular diseases, with a rising global incidence. Despite their clinical importance, the underlying pathogenesis of these diseases and the development of reliable non-invasive diagnostic tools remain inadequately understood. Accumulating evidence suggests that gut microbiota and its associated metabolites may play a crucial role in the development of kidney diseases via the gut-kidney axis. However, comprehensive studies integrating both microbiome and metabolomic data in IgAN and MN are still limited.

**Methods:**

In this study, we performed integrated metagenomic sequencing and untargeted metabolomic profiling to investigate alterations in gut microbial composition and systemic metabolic changes associated with IgAN and MN. Fecal samples were collected from 24 patients with IgAN, 20 patients with MN, and 17 healthy controls. Microbial diversity and composition were assessed using metagenomic analysis, while metabolic profiles were evaluated through untargeted LC -MS-based metabolomics. Multivariate statistical analyses and biomarker modeling were employed to identify discriminative features and evaluate diagnostic performance. Microbiota-metabolite correlation networks were constructed to explore potential mechanistic links.

**Results:**

Metagenomic analysis showed that both the IgAN and MN groups had significantly reduced α-diversity. Although β-diversity analysis did not reveal significant differences between the three groups, the IgAN and MN groups exhibited higher sample dispersion than the control group. Notably, both IgAN and MN patients showed a decrease in the abundance of certain specific microbial taxa. A total of 34 and 28 differentially abundant microbial species were identified in IgAN and MN, respectively, compared to healthy controls, with 16 taxa consistently downregulated in both disease groups. Notably, *Streptococcus oralis* was significantly enriched in the MN group, while *[Clostridium] innocuum* was markedly depleted. Metabolomic profiling identified 307 and 209 differentially abundant metabolites in IgAN and MN, respectively. Dipeptides (e.g., prolylleucine) were consistently upregulated, while the levels of certain short-chain fatty acids (SCFA) were reduced. Multivariate biomarker models demonstrated excellent diagnostic performance, achieving area under the curve (AUC) of 0.919 (IgAN vs. control), 0.897 (MN vs. control) and 0.912 (IgAN vs. MN), surpassing individual metabolite markers.

**Discussion:**

Our findings highlight significant alterations in gut microbial composition and systemic metabolite profiles in both IgAN and MN patients compared to healthy individuals. The consistent reduction in microbial diversity and SCFA-producing taxa, along with characteristic changes in metabolic signatures, supports the involvement of the gut-kidney axis in disease pathogenesis. The diagnostic models developed in this study provide promising non-invasive biomarkers for distinguishing IgAN and MN with high accuracy. These results contribute novel insights into the microbe-metabolite interplay in glomerular diseases and offer potential targets for future diagnostic and therapeutic strategies.

## 1 Introduction

Chronic kidney disease (CKD) is an increasingly severe global public health issue, characterized by a persistent decline in renal function, which may eventually progress to end-stage renal disease (ESRD). In recent years, the gut-kidney axis has garnered significant attention for its critical role in the pathogenesis of CKD. A growing body of research indicates that the gut microbiota plays an important role in the onset and progression of CKD. Bibliometric analyses have shown that from 2001 to 2022, studies related to the gut microbiota in the context of CKD have steadily increased, becoming a research hotspot. The mechanisms underlying this association and the potential for therapeutic intervention are being extensively explored (1). Current research primarily focuses on the impact of dysbiosis on disease progression, the potential efficacy of probiotic interventions, and the elucidation of microbial metabolic pathways. These findings provide a theoretical basis for better understanding CKD pathogenesis and exploring novel treatment strategies. Recent studies further confirm the therapeutic potential of specific bacterial strains in CKD. For instance, the abundance of *Lactobacillus johnsonii* has been found to be significantly reduced in CKD patients, with a positive correlation between its abundance and renal function. In animal models, supplementation with *L. johnsonii* effectively alleviated renal injury and fibrosis (2). This finding not only underscores the close relationship between gut microbiota and renal function, but also offers a new perspective for microbiota-based therapeutic strategies in CKD.

In addition to microbial dysbiosis, CKD patients often experience systemic metabolic abnormalities. In recent years, metabolomics has emerged as a key tool for elucidating physiological and pathological metabolic pathways, playing a crucial role in the identification of CKD-related biomarkers, clarification of metabolic mechanisms, and exploration of therapeutic pathways. Studies have shown that dysregulation in phospholipid metabolism, fatty acid oxidation, and amino acid metabolism is closely associated with CKD and renal fibrosis. For example, various phospholipid and glycerophospholipid metabolites are significantly elevated in patients with ESRD and remain high even after dialysis, potentially increasing the risk of cardiovascular and other complications (3). In animal models of CKD, disrupted phosphatidylcholine metabolism has been shown to regulate inflammatory responses and membrane remodeling through the phospholipase A₂ (PLA₂) pathway, potentially contributing to renal fibrosis (Wang et al., 2023). Moreover, diabetic kidney disease (DKD), a serious complication of diabetes, involves complex pathogenic mechanisms encompassing multiple metabolic and immune pathways. Studies have revealed that DKD patients exhibit gut microbiota dysbiosis and abnormal microbial metabolites, which are closely linked to renal inflammation and fibrosis (5). In terms of treatment, traditional Chinese medicine (TCM) formulations, such as the combination of Astragalus and Curcuma, have shown efficacy in improving renal function in CKD animal models by modulating amino acid, energy, and lipid metabolism pathways (6). Additionally, metabolomics-based research has identified a range of metabolites as potential biomarkers for the diagnosis, classification, and prognosis of CKD (7).

Immunoglobulin A nephropathy (IgAN) and membranous nephropathy (MN) are two common primary glomerular diseases (PGD), both of which can lead to chronic kidney injury and may progress to CKD or ESRD. IgAN is characterized by the abnormal deposition of immunoglobulin A (IgA) in the glomerular mesangial area. Its pathogenesis may involve both genetic and environmental factors, including infections, dietary antigens, microbiota alterations, and dysregulated mucosal immune responses (8). MN is a chronic autoimmune disease characterized by the deposition of immune complexes in the subepithelial region of the glomerular basement membrane, leading to glomerular injury (10, 9). Although the two diseases differ in their pathological mechanisms, both are closely associated with immune dysregulation and may be jointly influenced by the host microbiota and its metabolic products. More importantly, retrospective studies have shown that PGD remain the most common kidney diseases in China, with IgAN having the highest prevalence, followed by MN. Furthermore, the incidence of MN is increasing (12–14). At present, the diagnosis of both diseases primarily relies on renal biopsy. However, this invasive procedure increases the physical burden and psychological stress on patients, thereby limiting its application in early screening and large-scale studies. Therefore, current research efforts are focused on identifying new biomarkers with the aim of achieving earlier and more accurate diagnosis and prediction (15–18). Therefore, IgAN and membranous nephropathy MN as research subjects not only helps to uncover their respective pathogenic mechanisms but also provides an opportunity to explore their potential shared features. Meanwhile, given that the diagnosis of both diseases currently relies heavily on renal biopsy, identifying non-invasive biomarkers has become an urgent need for achieving early diagnosis and precise monitoring.

An increasing body of research suggests that gut microbiota plays a crucial role in the onset and progression of IgAN and MN, with its dysbiosis potentially impacting kidney health through various mechanisms (19–20). Studies have shown that alterations in the gut microbiota may mediate the production of galactose-deficient IgA1 (gd-IgA1) by affecting the mucosal immune system and intestinal barrier function, thereby promoting the onset and progression of IgAN (22–24). Several studies have investigated microbiome characteristics in patients with IgAN and MN. For instance, research has demonstrated that the gut microbiome of MN patients exhibits significantly lower diversity and richness compared to healthy individuals, highlighting the potential value of gut microbiota as a non-invasive diagnostic tool for MN (25, 26). In addition, some studies have preliminarily revealed the potential role of the gut-kidney axis in IgAN patients (28–27), Wu et al. established a relationship network between the microbiota, fecal metabolites, and serum metabolites in IgAN, identifying six key metabolites (including bilirubin, trimethoprim, stearamide, phenylalanine, cis-9,10-epoxystearic acid, and PE lyso 17:0) that highlight the metabolic network connection between the gut and blood (27). However, research on the pathological state of MN remains limited, and comparative studies between MN and IgAN are lacking. Notably, these studies are still based on 16S rRNA sequencing, which has limitations in resolution and functional annotation, and thus fails to fully uncover the complex interplay between the microbiota and metabolites.

By integrating metagenomics and metabolomics, this approach can provide higher-resolution insights into the composition and functionality of the gut microbiome, offering a deeper understanding of how microbial metabolites impact kidney health. Through the integration of these two omics technologies, this study will comprehensively analyze the specific microbiome characteristics and related metabolic changes in IgAN and MN. Furthermore, based on these findings, potential biomarkers will be identified, providing new strategies for early diagnosis, accurate prognosis, and the development of personalized treatment approaches.

## 2 Materials and methods

### 2.1 Study subjects and sample collection

A total of 61 subjects from Guang’an First People’s Hospital, Sichuan Province, China, were enrolled in this study. The participants were categorized into three groups: healthy controls (control, *n* = 17), patients with IgA nephropathy (IgAN, *n* = 24), and patients with membranous nephropathy (MN, *n* = 20). Patients in the IgAN and MN groups were diagnosed via renal biopsy and confirmed through direct immunofluorescence, light microscopy, and electron microscopy evaluations, while secondary glomerular diseases were excluded. None of the patients had received glucocorticoids, immunosuppressants, or other therapeutic interventions prior to diagnosis, although a subset of patients had used angiotensin receptor blockers (ARB) before sample collection to reduce urinary protein excretion. The healthy control group was confirmed through physical examination to have no history of kidney disease, normal liver and kidney function, and no abnormalities in urine and stool routine tests. Detailed inclusion and exclusion criteria for IgAN and MN are provided in Appendix 1. Basic clinical information, including age, sex, and renal function indicators, was collected for all participants. Fecal samples were collected in sterile sampling tubes and stored at −80°C within 2 h for subsequent metagenomic and metabolomic analyses. This study was approved by the Ethics Committee of Guang’an People’s Hospital (permit number: 2024–045).

### 2.2 Metagenomic sequencing and data processing

DNA was extracted from fecal samples and its integrity, size, and concentration were measured using the Agilent 5,400 (Agilent, United States). Library construction was then performed, including DNA fragmentation, end repair, 3′ A-tailing, adapter ligation, fragment selection, and PCR amplification. The libraries were evaluated for quality. Qualified libraries were sequenced using the NovaSeq 6,000 platform (Illumina) with paired-end 150 base pair (PE150) reads. Data quality control was performed using fastp to remove low-quality reads and adapter contamination, generating clean data. Clean reads were aligned to the human reference genome (GCF_000001405.40) using Bowtie2 (v2.5.1) to remove host contamination. Subsequently, species identification was performed using Kraken2 (v2.1.3) combined with the microbial database (PlusPF, 2023-10-09), and taxonomic abundance was re-assigned using Bracken (v2.9). The resulting microbial abundance table was then compiled. The metagenomic clean reads were assembled individually using MEGAHIT (v1.2.9). Gene prediction was performed using Prodigal (v2.6.3), followed by redundancy removal using CD-HIT (v4.8.1) with 95% similarity and 90% coverage. The nucleotide sequences were then translated into amino acid sequences. Kyoto Encyclopedia of Genes and Genomes (KEGG) orthologous gene annotation was carried out using EggNOG-mapper (v2.1.12) in conjunction with the eggNOG database. Finally, non-redundant gene abundance was calculated using Salmon (v1.10.2) and normalized to transcripts per million (TPM).

### 2.3 Untargeted metabolomics analysis and data processing

Fecal samples (100 mg) were added to 500 μL of 80% methanol aqueous solution (Methanol: LC–MS Grade, Thermo Fisher, United States; Water: LC–MS Grade, Merck, Germany), vortexed, and incubated in an ice bath for 5 min. The samples were then centrifuged at 15,000 g and 4°C for 20 min. The supernatant was collected and diluted to a final methanol concentration of 53%. After a second centrifugation, the supernatant was used for LC–MS analysis. QC samples were prepared by pooling equal volumes of extracts from each fecal sample and were used to assess the stability and reproducibility of the metabolomics analysis platform. Blank samples were prepared by replacing the fecal material with 53% methanol–water and processed using the same pretreatment procedure, serving to eliminate background ions. LC–MS analysis was performed using a Q Exactive™ HF/Q Exactive™ HF-X mass spectrometer (Thermo Fisher, Germany) coupled with a Vanquish UHPLC system (ThermoFisher, Germany). A Hypersil Goldcolumn (100 × 2.1 mm, 1.9 μm, Thermo Fisher, USA) was used for chromatographic separation, employing gradient elution with a column temperature of 40°C and a flow rate of 0.2 mL/min. Mass spectrometry was conducted using an electrospray ionization (ESI) source in both positive and negative ion modes, with a scanning range of m/z 100–1,500. The raw data were imported into Compound Discoverer 3.3 (CD 3.3) software for preliminary processing. Each metabolite was initially screened based on parameters such as retention time and mass-to-charge ratio (m/z), with peak area correction performed using the first QC sample to enhance the accuracy of metabolite identification. The mass tolerance was set to 5 ppm, and the signal intensity deviation was set to 30%, with a minimum intensity threshold defined. Additional parameters such as adduct ions were considered for peak extraction and peak area quantification. Based on this, target ion information was integrated and molecular formulas were predicted using molecular and fragment ion peaks. Metabolite identification was conducted by matching against the mzCloud,^1^ mzVault and Masslist databases, while background ions were removed based on blank sample data. The raw quantitative data were normalized using the following formula: Sample raw intensity / (Total metabolite intensity in the sample / Total metabolite intensity in QC1 sample), yielding relative peak areas. Metabolites with a coefficient of variation (CV) greater than 30% in QC samples were excluded. The final results included both metabolite identification and relative quantification. Identified metabolites were further annotated for biological significance using KEGG database,^2^ HMDB database^3^ and LIPIDMaps databases.^4^

### 2.4 Statistical analysis

Statistical analyses in this study were conducted using R software. Clinical data analyses were performed using the tableone (v0.13.2) R package (31). Categorical variables were analyzed using the Chi-square test, whereas continuous variables were compared between two groups using the t-test. Metagenomic microbial composition analyses were performed using the vegan (v2.6–10) R package (32). Bray–Curtis distance matrices were constructed to assess differences in microbial community composition among groups, and statistical significance was evaluated using permutational multivariate analysis of variance (PERMANOVA) implemented via the adonis2 function. Furthermore, beta diversity dispersion was computed using the betadisper function, and the significance was tested using permutest. The Alpha diversity index was calculated using the vegan package. Differential species screening was performed using the microeco (v1.9.1) R package (30) based on the Linear Discriminant Analysis Effect Size (LEfSe) method, employing Linear Discriminant Analysis (LDA) to select significant features with LDA > 2 and *p* < 0.05. Metagenomic KEGG Orthology (KO) gene functional analysis was conducted using the MicrobiotaProcess (v1.10.0) R package, with mp_diff_analysis for differential gene identification, followed by functional enrichment analysis based on the KEGG database using the clusterProfiler (v4.8.1) R package. Metabolite data analysis was performed using the MetaboAnalystR (v3.0.3) R package (34). Median normalization was applied to eliminate batch effects, followed by auto-scaling to enhance data consistency. Principal Component Analysis (PCA) and Orthogonal Partial Least Squares Discriminant Analysis (OPLS-DA) were then used for classification modeling. Model performance was evaluated using R^2^X, R^2^Y, and Q^2^ to assess goodness-of-fit and predictive ability. In addition, Variable Importance in Projection (VIP) scores were calculated to evaluate the contribution of individual metabolites to the model. Differential metabolite analysis was performed by calculating the fold change (FC) and assessing significance using the Student’s t-test. Metabolites with FC > 1.5 or FC < 0.667, VIP > 1, and *p* < 0.05 were considered as significant differential metabolites. Metabolic pathway analysis was conducted using the pathway analysis module of MetaboAnalyst 6.0.^5^ To assess the classification performance of the metabolic features, we constructed a Logistic Regression model and employed 1,000 bootstrap resampling iterations to calculate the Receiver Operating Characteristic (ROC) curve, along with the Area under the Curve (AUC), to evaluate the model’s stability and accuracy. First, metabolites with *p* < 0.05 and AUC > 0.8 were selected as potential biomarkers. Then, metabolites with *p* < 0.05 and AUC > 0.7 were included in Lasso regression for feature selection, and 10-fold cross-validation was used to optimize the model. Finally, metabolites with a Variance Inflation Factor (VIF) < 10 were chosen to construct a multivariable Logistic Regression model. Additionally, Spearman’s rank test was used to calculate the correlation between microbiota and metabolite concentrations, aiming to explore their potential interactions. All statistical analyses were performed with a significance threshold of *p* < 0.05.

## 3 Results

### 3.1 Clinical characteristics

The age of the IgAN group was significantly different compared to the MN group (*p* < 0.05), while there were no statistically significant differences in gender, hypertension, or diabetes prevalence across the three groups (*p* > 0.05). In terms of kidney function-related indicators, the IgAN group showed a significant decrease in estimated Glomerular Filtration Rate (eGFR; *p* = 0.022) and a significant increase in serum creatinine levels (*p* = 0.006) compared to the control group, whereas no significant differences were observed in these two indicators for the MN group (*p* = 0.245/0.19). In addition, there were no statistically significant differences in urea levels among the three groups (*p* > 0.05). Both the IgAN and MN groups had significantly higher uric acid levels compared to the control group (*p* = 0.02/0.023). Furthermore, the MN group had significantly higher Low-Density Lipoprotein Cholesterol (LDL-C) levels than both the control group (*p* = 0.005) and the IgAN group (*p* = 0.009), while serum albumin levels were significantly lower than those in the other two groups (*p* < 0.001). There were no significant differences in LDL-C and serum albumin levels between the IgAN group and the control group (*p* = 0.364/0.21) (Table 1).

**Table 1:** Clinical characteristics.

Parameters	control (*n* = 17)	IgAN (*n* = 24)	MN (*n* = 20)	*p* value (control vs. IgAN/MN)	*p* value (IgAN vs. MN)
Age (yr), mean±SD	52.44 ± 19.55	43.96 ± 12.25	53.75 ± 9.08	0.092/0.79	0.005
Male, *n* (%)	10 (55.6)	9 (37.5)	12(60.0)	0.395/1	0.236
Hypertension, *n* (%)	3(16.7)	11 (45.8)	9 (45.0)	0.098/0.127	1
Diabetes, *n* (%)	1 (5.6)	0 (0.0)	1 (5.0)	0.884/1	0.926
eGFR (ml/min per 1.73 m^2^), mean±SD	97.96 ± 19.73	76.85 ± 33.25	90.31 ± 20.09	0.022/0.245	0.121
Blood Creatinine (umol/L), mean±SD	67.44 ± 20.48	102.53 ± 47.44	81.00 ± 38.33	0.006/0.19	0.11
Urea (mmol/l), mean±SD	6.43 ± 1.80	6.11 ± 1.93	7.28 ± 2.58	0.585/0.252	0.092
Uric acid (μmol/L), mean±SD	307.86 ± 114.99	403.45 ± 118.14	399.46 ± 107.21	0.02/0.023	0.908
LDL-C (mmol/L), mean±SD	2.96 ± 1.38	3.39 ± 1.38	4.83 ± 1.98	0.364/0.005	0.009
Albumin Blood (g/L), mean±SD	43.46 ± 3.82	40.92 ± 6.82	29.22 ± 7.93	0.21/<0.001	<0.001

### 3.2 Metagenomics analysis

**Figure 1:**
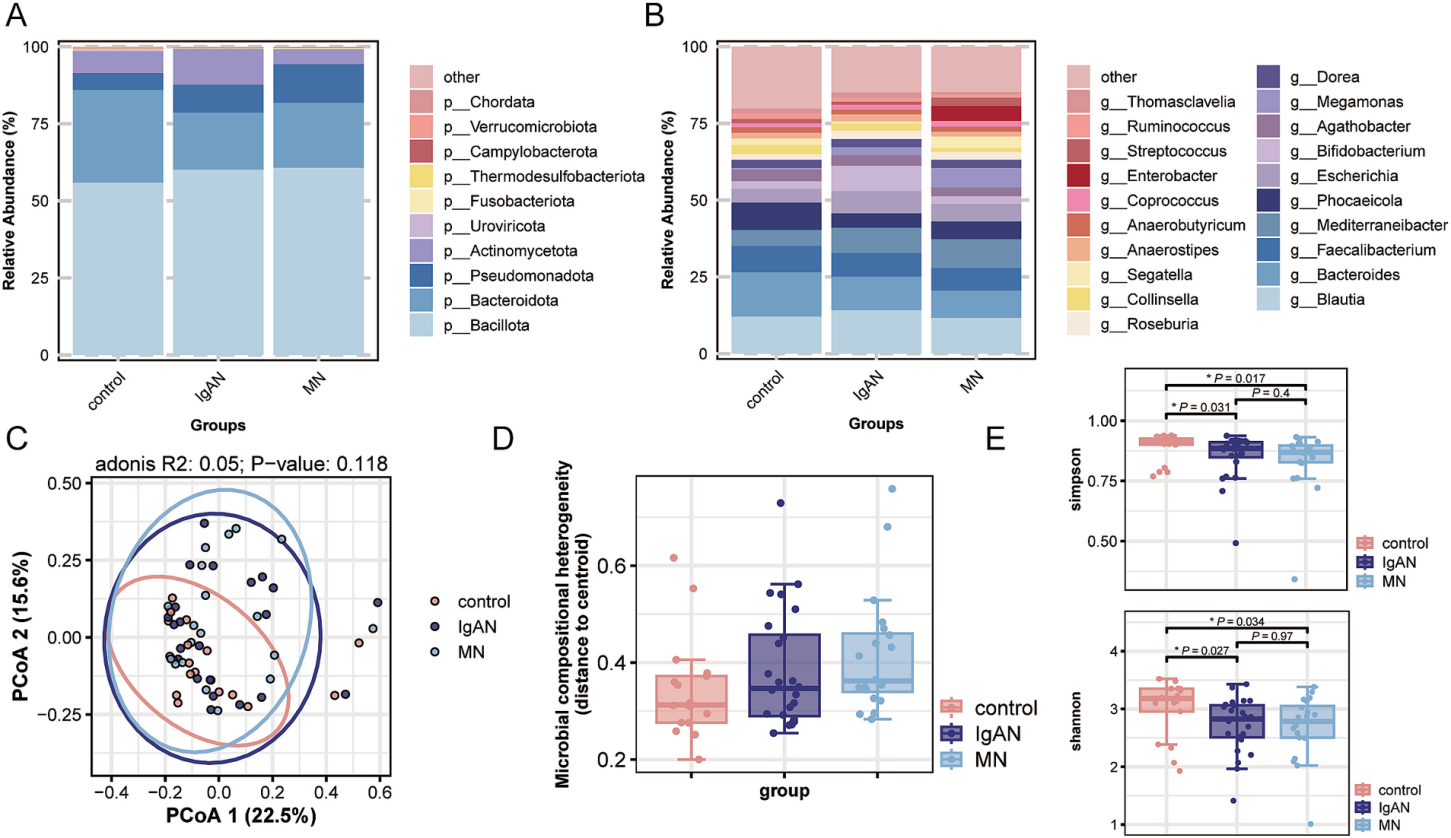
Metagenomic species annotation. **(A)** Stacked bar chart showing the average relative abundance of the top 10 phyla in each group, reflecting the phylum-level microbiota composition across IgAN, MN and controls; **(B)** Stacked bar chart showing the average relative abundance of the top 20 genera in each group, reflecting the genus-level microbiota composition across IgAN, MN and controls; **(C)** PCoA based on genus-level microbial composition, with group differences assessed by PERMANOVA. The adonis results showed R^2^ = 0.05 and *p*-value = 0.118; **(D)** Box plot of *β*-diversity (Beta-dispersion) at the genus level (no significant differences between groups); **(E)** Box plot of *α*-diversity (Simpson and Shannon index) at the genus level.

At the phylum level, Bacillota, Bacteroidota, Pseudomonadota and Actinomycetota were the dominant phyla in all three groups. Among them, the relative abundance of Bacteroidota was lower in the IgAN group, while the relative abundance of Actinomycetota was higher; the control group showed a trend of lower relative abundance of Pseudomonadota (Figure 1A). Figure 1B displays the top 20 genera by relative abundance in the three groups. At the genus level, we calculated *β*-diversity using Bray-Curtis distance and visualized microbial compositional differences between samples through Principal Coordinate Analysis (PCoA). PERMANOVA results indicated no significant differences between the IgAN, MN, and control groups (*p* > 0.05; Figure 1C). Additionally, β-diversity analysis assessed the variation in sample diversity within each group. The results indicated that the control group had lower dispersion, while the IgAN and MN groups exhibited higher dispersion. However, this difference was not statistically significant (*p* > 0.05; Figure 1D). In terms of *α*-diversity, both the IgAN and MN groups showed a significant decrease in Simpson and Shannon indices compared to the control group, while no significant difference was observed between the two disease groups (Figure 1E).

**Figure 2:**
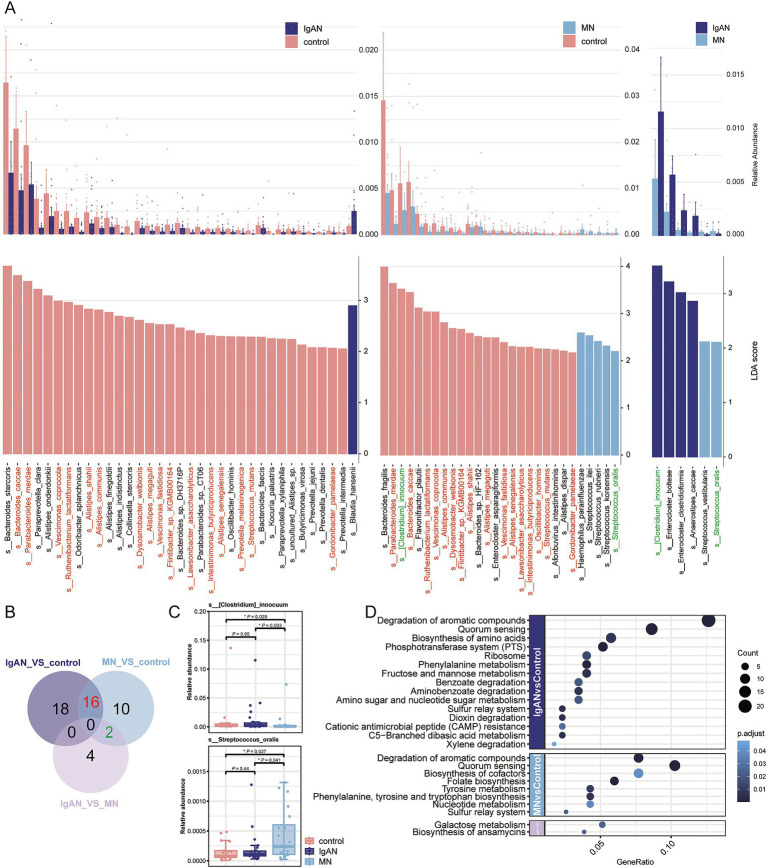
Metagenomic differential analysis. **(A)** LEfSe analysis results showing significantly different taxa among the IgAN vs. control, MN vs. control, and IgAN vs. MN groups (LDA score > 2, *p* < 0.05). The upper panel displays the relative abundance bar plots, while the lower panel shows the corresponding LDA scores. **(B)** Venn diagram summarizing the overlap of significantly different taxa among the three pairwise comparisons. Shared taxa between IgAN and MN (vs. controls) are highlighted in red, suggesting potentially common microbial alterations. Taxa shared between MN vs. control and IgAN vs. MN are marked in green. These taxa are consistently color-coded in panel A for cross-reference; **(C)** Box plots showing the relative abundance of two representative taxa with disease-specific alterations: *Streptococcus oralis*, significantly enriched in MN, and *[Clostridium] innocuum*, significantly depleted in MN, compared to controls and IgAN. **(D)** Bubble plot illustrating KEGG pathway enrichment analysis of differentially abundant KO genes. Bubble size represents the number of enriched KO terms within each pathway, while color intensity reflects the statistical significance of enrichment (*p* value), revealing distinct functional disturbances in the gut microbiome across disease groups.

The LEfSe algorithm was applied to identify species-level microbial differences among the IgAN, MN, and control groups. The results revealed that 34 differential microorganisms were identified between the IgAN group and the control group, 28 between the MN group and the control group, and 6 between the IgAN and MN groups (LDA score > 2.0, *p* < 0.05; Figure 2A; Supplementary Table 2). Among these differential species, 16 exhibited significantly higher relative abundance in the control group compared to both disease groups. In addition, two species showed distinct alterations in the MN group relative to both the IgAN and control groups (Figure 2B). *[Clostridium] innocuum* displayed the lowest relative abundance in the MN group, whereas *Streptococcus oralis* showed the highest relative abundance in the MN group (Figure 2C). In the comparison between the IgAN group and the control group, a total of 15 significantly enriched pathways were identified. These mainly involved carbohydrate and amino acid metabolism, including degradation of aromatic compounds (ko01220), phenylalanine metabolism (ko00360), C5-branched dibasic acid metabolism (ko00660), fructose and mannose metabolism (ko00051), and amino sugar and nucleotide sugar metabolism (ko00520). In the MN group compared to the control group, 8 pathways were significantly enriched, such as quorum sensing (ko02024), folate biosynthesis (ko00790), and tyrosine metabolism (ko00350). Additionally, comparison between the IgAN and MN groups revealed significant enrichment of biosynthesis of ansamycins (ko01051) and galactose metabolism (ko00052) in the IgAN group, indicating distinct microbial functional signatures between the two diseases (Figure 2D).

### 3.3 Fecal metabolomics analysis

**Figure 3:**
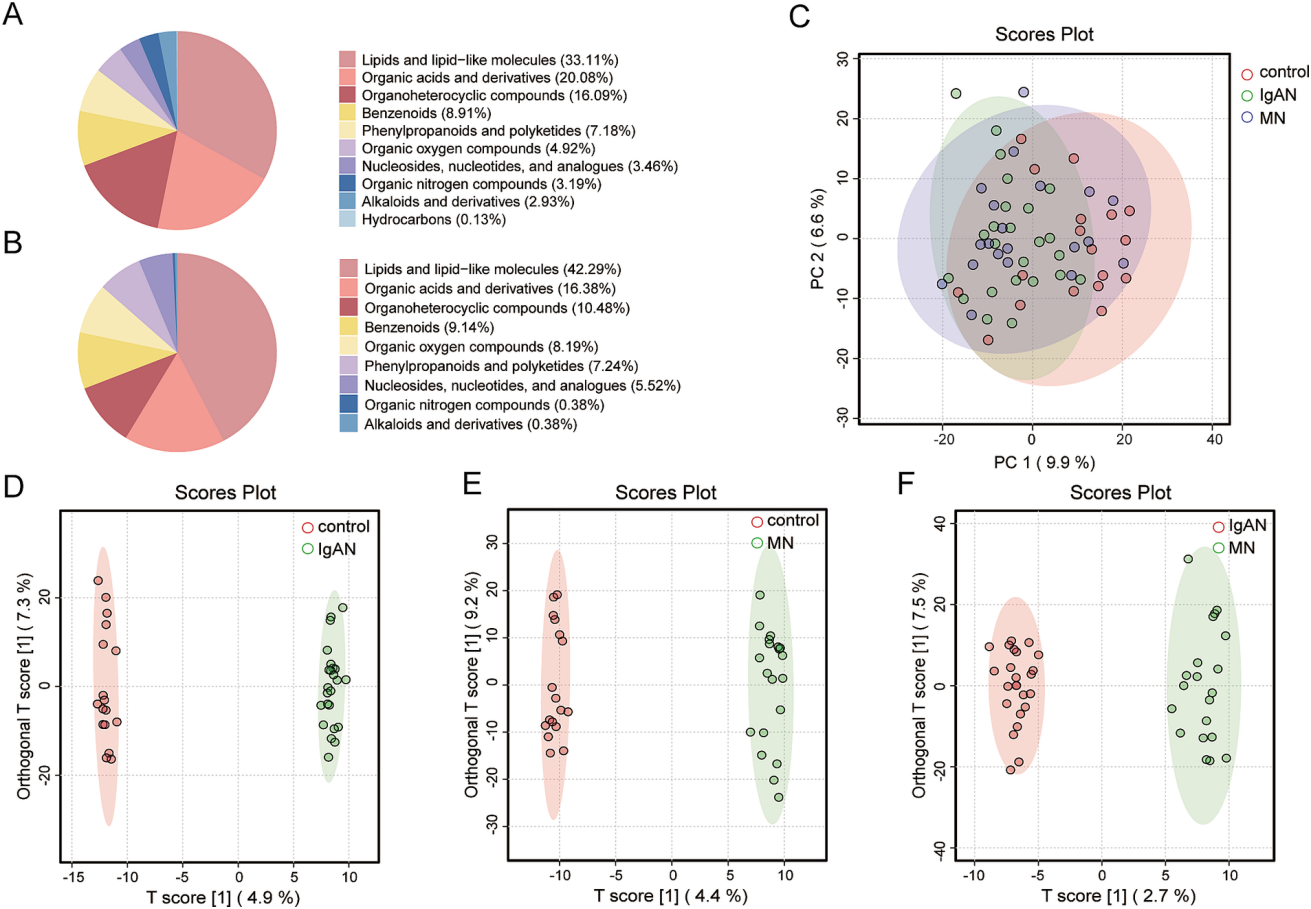
Metabolite classification and multivariate statistical analysis. **(A)** Pie chart of Class I metabolite classification in positive ion mode; **(B)** Pie chart of Class I metabolite classification in negative ion mode; **(C)** PCA score plot for IgAN, MN and control groups. The figure illustrates the distribution of samples among the IgAN, MN and control groups, with IgAN represented in green, MN in blue, and controls in red; **(D)** OPLS-DA score plot for IgAN vs. control. This model was employed to identify metabolite-level differences between the IgAN and control groups. The model’s goodness-of-fit metrics were *R*^2^X = 0.198 and *R*^2^Y = 0.998, with a predictive ability of Q^2^ = 0.478; **(E)** OPLS-DA score plot for MN vs. control. This model was employed to identify metabolite-level differences between the MN and control groups. The model’s goodness-of-fit metrics were *R*^2^X = 0.232 and *R*^2^Y = 0.995, with a predictive ability of *Q*^2^ = 0.32; **(F)** OPLS-DA score plot for IgAN vs. MN. This model was employed to identify metabolite-level differences between the IgAN and MN groups. The model’s goodness-of-fit metrics were *R*^2^X = 0.142 and *R*^2^Y = 0.981, with a predictive ability of *Q*^2^ = 0.156.

A total of 1,347 and 782 metabolites were identified in the positive and negative ion modes, respectively, across 61 fecal samples (Supplementary Table 3). The chemical classification of the identified metabolites was statistically analyzed, and pie charts for Metabolite Class I were created to reflect the classification of the detected metabolites and the count of metabolites in each category. In positive ion mode, the five most abundant classes were Lipids and lipid-like molecules, Organic acids and derivatives, Organoheterocyclic compounds, Benzenoids, and Phenylpropanoids and polyketides (Figure 3A). In negative ion mode, the top classes were Lipids and lipid-like molecules, Organic acids and derivatives, Organoheterocyclic compounds, Benzenoids, and Organic oxygen compounds (Figure 3B). Metabolites from both ion modes were combined for PCA to assess overall differences among the IgAN, MN, and control groups. The PCA score plot showed significant differences between the IgAN and control groups (*p* = 0.001) and between the MN and control groups (*p* = 0.012), while no significant difference was observed between the IgAN and MN groups (*p* = 0.565; Figure 3C). Further OPLS-DA model analysis also confirmed that the disease groups were clearly separated from the healthy controls, indicating significant changes in the metabolic state of both IgAN and MN patients. The R2X and R2Y values of the model assessed the explained variance of the independent and dependent variable matrices, while Q2 represented the model’s predictive ability. Different clustering results were observed in the comparisons between the groups (IgAN vs. control: *R*^2^X = 0.198, *R*^2^Y = 0.998, *Q*^2^ = 0.478; MN vs. control: *R*^2^X = 0.232, *R*^2^Y = 0.995, *Q*^2^ = 0.32; IgAN vs. MN: *R*^2^X = 0.142, *R*^2^Y = 0.981, *Q*^2^ = 0.156; Figures 3D–F).

**Figure 4:**
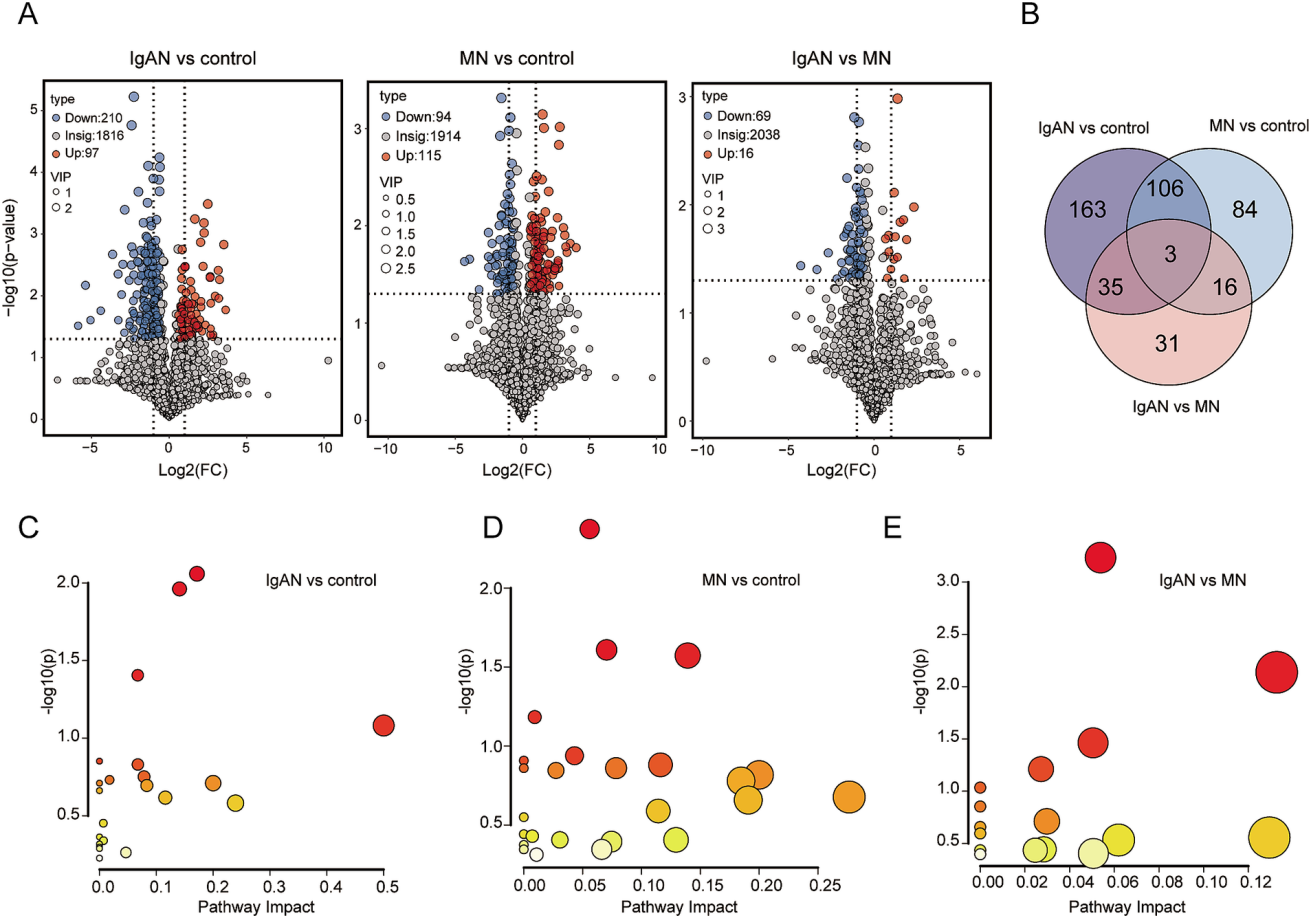
Differential metabolite analysis and enriched metabolic pathway analysis. **(A)** Volcano plots of metabolites between IgAN vs. control, MN vs. control and IgAN vs. MN. The highlighted points in the figure represent significantly differential metabolites selected based on the criteria of Variable Importance in Projection (VIP) > 1.0, log2 fold change (Log2 FC) ≥ 1 and statistical significance with *p*- value < 0.05. Blue represents downregulated differential metabolites, and red represents upregulated differential metabolites; **(B)** Venn diagram of differential metabolites. The numbers in the diagram represent the number of overlapping differential metabolites between groups or the unique differential metabolites in each group; **(C)** Metabolic pathway topology map for IgAN vs. control. The y-axis represents the -log(p) value from pathway enrichment analysis, and the x-axis represents the impact factor from topology analysis; **(D)** Metabolic pathway topology map for MN vs. control; **(E)** Metabolic pathway topology map for IgAN vs. MN.

Differential metabolite screening was conducted using fold change (FC), variable importance in projection (VIP), and *p*-value criteria (Figure 4A). Between the IgAN and control groups, 307 significantly differential metabolites were identified, with 97 metabolites significantly upregulated and 210 metabolites significantly downregulated. Between the MN and control groups, 209 significantly differential metabolites were identified, with 115 metabolites significantly upregulated and 94 metabolites significantly downregulated. Between the IgAN and MN groups, 85 significantly differential metabolites were identified, with 16 metabolites significantly upregulated and 69 metabolites significantly downregulated (Supplementary Table 4). The Venn diagram of differential metabolites shows the number of overlapping and unique differential metabolites between each pair of groups (Figure 4B). There are 163 unique differential metabolites between IgAN and control; 84 unique differential metabolites between MN and control; and 31 unique differential metabolites between IgAN and MN. The three groups shared three common differential metabolites. We imported the selected differential metabolites into MetaboAnalyst 6.0 for pathway analysis. Based on the topological structure of the metabolic pathways, we calculated the significance and impact factor of each pathway (Figures 4C–E). The metabolic pathways enriched in the IgAN and control groups (*p* < 0.05) included Steroid hormone biosynthesis, Tyrosine metabolism, and Glycerophospholipid metabolism. The metabolic pathways enriched in the MN and control groups (*p* < 0.05) included *β*-Alanine metabolism, Purine metabolism, and Histidine metabolism. Between the IgAN and MN groups (*p* < 0.05), the enriched pathways included purine metabolism, pyrimidine metabolism, and alanine, aspartate, and glutamate metabolism. Detailed metabolite levels and impact scores are provided in Supplementary Table 5.

### 3.4 Metabolite biomarker analysis

**Figure 5:**
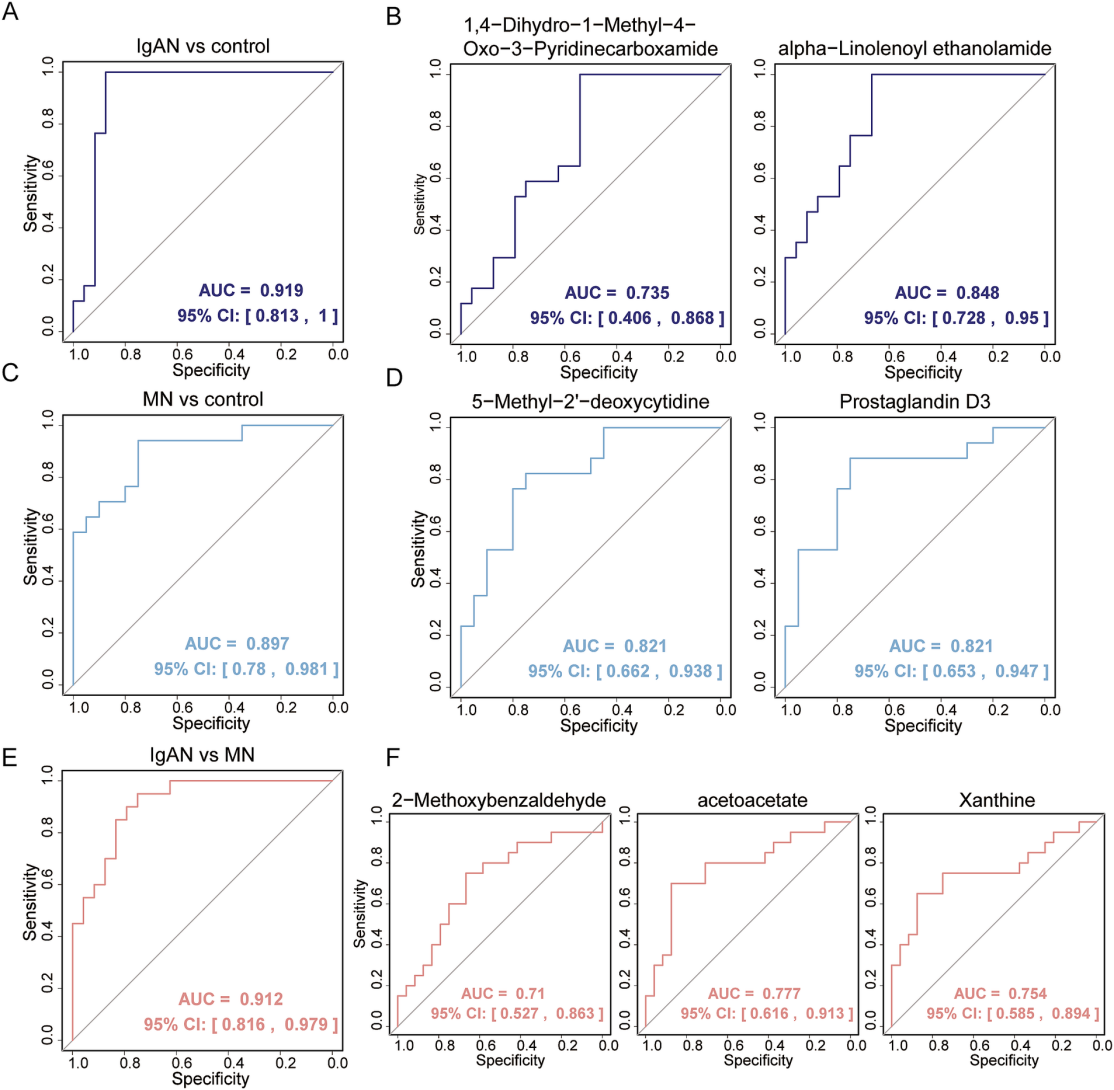
Evaluation of fecal metabolic biomarkers in distinguishing disease patients from healthy controls and in differentiating between two kidney different diseases. **(A)** ROC analysis of multivariable biomarker combinations for distinguishing IgAN from control, with an AUC value of 0.919; **(B)** ROC analysis of univariable biomarkers for distinguishing IgAN from control, including 1,4-Dihydro-1-Methyl-4-Oxo-3-Pyridinecarboxamide and alpha-Linolenoyl ethanolamide, with AUC values of 0.735 and 0.848, respectively; **(C)** ROC analysis of multivariable biomarker combinations for distinguishing MN from control, with an AUC value of 0.897; **(D)** ROC analysis of univariable biomarkers for distinguishing MN from control, including 5-Methyl-2′-deoxycytidine and Prostaglandin D3, both with AUC values of 0.821; **(E)** ROC analysis of multivariable biomarker combinations for distinguishing IgAN from MN, with an AUC value of 0.912; **(F)** ROC analysis of univariable biomarkers for distinguishing IgAN from MN, including 2-Methoxybenzaldehyde, acetoacetate and Xanthine, with AUC values of 0.71, 0.777 and 0.754, respectively.

To reduce the impact of external factors (such as vitamin supplements, medications, smoking, and diet) on metabolite levels, exposomics and vitamin metabolites were excluded from the analysis. Univariate logistic regression analysis was first conducted to identify metabolites with *p* < 0.05 and AUC > 0.8 as candidate biomarkers (Supplementary Table 6). In IgAN, a total of 14 potential biomarkers were identified, including alpha-Linolenoyl ethanolamide, Oleoyl ethanolamide and N-Oleoyl Glycine. In MN, three potential biomarkers were identified: 5-Methyl-2′-deoxycytidine, Prostaglandin D3 and N-Acetyl-D-tryptophan. For distinguishing between IgAN and MN, 4-Methyl-2-Oxopentanoic Acid was identified as a potential biomarker. All of these biomarkers demonstrated good diagnostic performance. Further screening of metabolites with AUC > 0.7 revealed that 1,4-Dihydro-1-Methyl-4-Oxo-3-Pyridinecarboxamide and alpha-Linolenoyl ethanolamide, when considered as univariate biomarkers for IgAN, had AUC values of 0.735 and 0.848, respectively. However, the combination of these two metabolites yielded an AUC of 0.919 [95% confidence interval (CI), 0.813–1.00], with a sensitivity of 1 and specificity of 0.875 (Figures 5A,B). For MN, 5-Methyl-2′-deoxycytidine and Prostaglandin D3, as univariate biomarkers, both had AUC values of 0.821. The combination of these two metabolites resulted in an AUC of 0.897 [95% confidence interval (CI), 0.780–0.981], with a sensitivity of 0.941 and specificity of 0.75 (Figures 5C,D). For distinguishing IgAN and MN, 2-Methoxybenzaldehyde, acetoacetate, and Xanthine as univariate biomarkers had AUC values of 0.710, 0.777, and 0.754, respectively. The combination of these three metabolites resulted in an AUC of 0.912 [95% confidence interval (CI), 0.816–0.979], with a sensitivity of 0.95 and specificity of 0.75 (Figures 5E,F). These results demonstrate that the combination of biomarkers significantly improved the predictive ability of the model.

### 3.5 Correlation analysis between microbiota and metabolites

**Figure 6:**
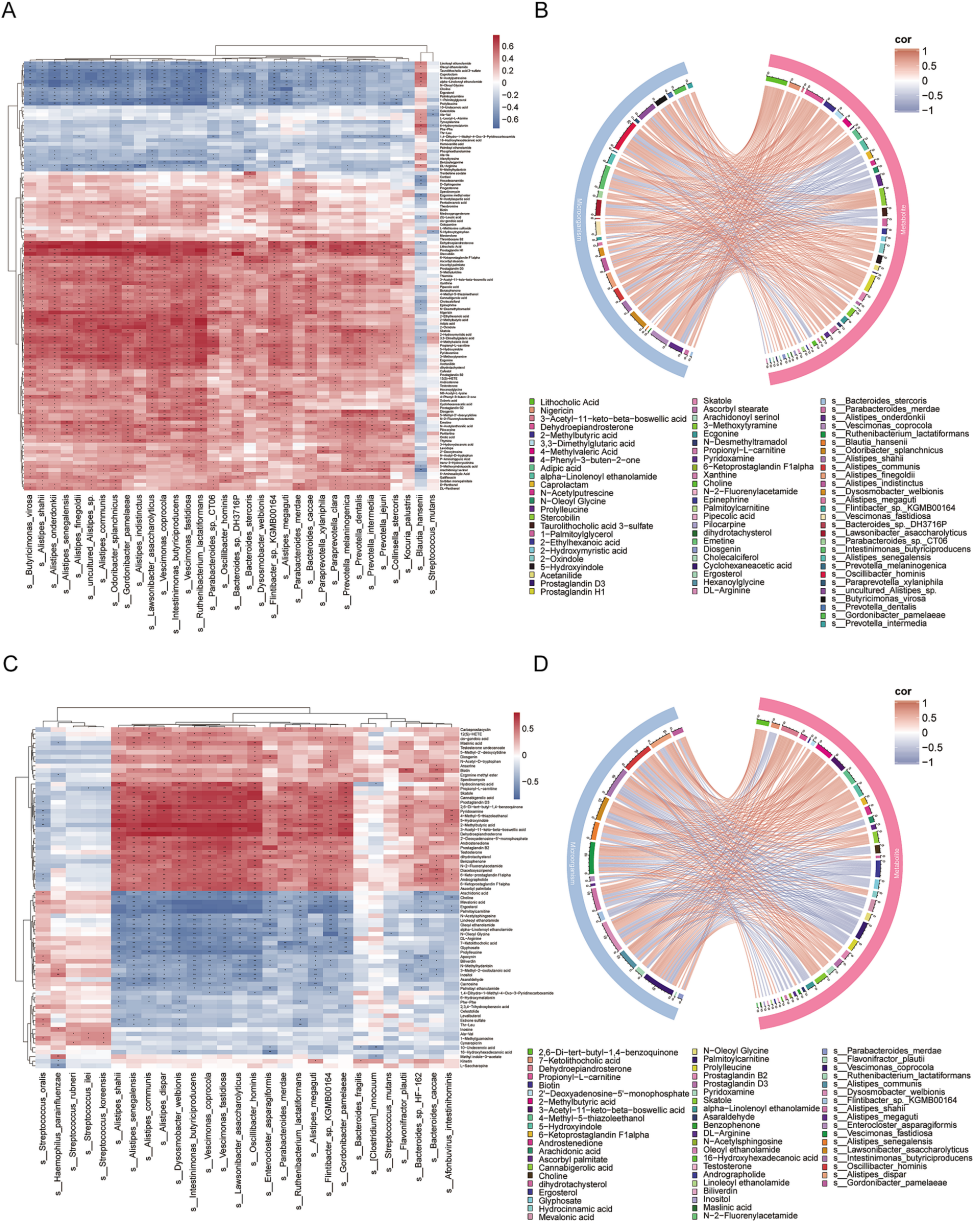
Correlation analysis between differentially abundant microorganisms and differentially abundant metabolites. **(A)** Heatmap of correlations between differentially abundant microorganisms and differentially abundant metabolites in IgAN vs. control. The colors in the heatmap represent the strength and direction of correlations, with red indicating positive correlations and blue indicating negative correlations. Statistical significance of the correlations is indicated by asterisks: an asterisk ‘*’ indicates 0.01 < *p* < 0.05, while two asterisks ‘**’ indicate *p* < 0.01. No significance is marked with an empty space; **(B)** Chord diagram of the correlation between differentially abundant microorganisms and differentially abundant metabolites in IgAN vs. control, based on FDR-corrected significance (*p* < 0.05) and strong correlation (|r| > 0.6). The chord colors indicate the strength and direction of correlations, with red representing positive correlations and blue representing negative correlations, thereby facilitating the identification of key microbe-metabolite interaction pairs; **(C)** Heatmap of correlations between differentially abundant microorganisms and differentially abundant metabolites in MN vs. control; **(D)** Chord diagram of the correlation between differentially abundant microorganisms and differentially abundant metabolites in MN vs. control, based on FDR-corrected significance (*p* < 0.05) and strong correlation (|r| > 0.6).

Spearman correlation analysis was used to assess the relationship between the microbiota and metabolites (Figures 6A,C). In IgAN, *Oscillibacter hominis* was significantly positively correlated with metabolites such as 2-Methylbutyric acid, 4-Methylvaleric Acid, Adipic acid, Ecgonine and Prostaglandin H1, and significantly negatively correlated with metabolites such as Taurolithocholic acid 3-sulfate, Prolylleucine, N-Oleoyl Glycine and 1-Palmitoylglycerol. *Vescimonas coprocola* was significantly positively correlated with metabolites like Stercobilin, Prostaglandin H1, Dehydroepiandrosterone and 2-Methylbutyric acid, and significantly negatively correlated with metabolites like N-Acetylputrescine and 1-Palmitoylglycerol. *Intestinimonas butyriciproducens* was significantly positively correlated with metabolites such as Diosgenin, Pilocarpine, Stercobilin and N-2-Fluorenylacetamide, and significantly negatively correlated with Prolylleucine (Figure 6B). In MN, *Intestinimonas butyriciproducens* was significantly positively correlated with metabolites like Cannabigerolic acid, Dehydroepiandrosterone, 2,6-Di-tert-butyl-1,4-benzoquinone and Skatole, and significantly negatively correlated with metabolites such as Mevalonic acid, Palmitoylcarnitine, Ergosterol and Arachidonic acid (Figure 6D).

## 4 Discussion

In this study, we analyzed the metagenomic and metabolomic features of IgAN and MN patients compared to healthy individuals.

### 4.1 Gut microbiota dysbiosis in IgAN and MN

The metagenomic results indicated that microbial diversity, community structure and the abundance of specific microbial taxa in the IgAN and MN groups were altered, suggesting that the gut microbiota in these two kidney disease patient groups has distinct compositional characteristics. In the β-diversity analysis, we observed greater dispersion in the IgAN and MN groups compared to the control group, indicating higher variability in microbial composition among individuals within these two groups. This increased dispersion could be influenced by various factors, such as the progression of the disease (25). Additionally, in the *α*-diversity analysis, we found a decrease in the Simpson and Shannon index at the genus level in both the IgAN and MN groups. It is noteworthy that several studies have already reported significant differences in α-diversity between IgAN patients and healthy controls (35–37).

At the species level, the relative abundance of certain specific microbial taxa was significantly different between IgAN and MN patients. For example, *Bacteroides fragilis* was enriched in the healthy control group but significantly decreased in the MN group. Studies have shown that *Bacteroides fragilis* alleviates kidney fibrosis in mice by reducing lipopolysaccharide (LPS) levels and increasing 1,5-anhydroglucitol (1,5-AG) levels. As an activator of TGR5, 1,5-AG can inhibit oxidative stress and inflammation, thereby alleviating kidney fibrosis (38). Additionally, *Blautia hansenii* was enriched in the IgAN group. Studies have found that changes in the abundance of bacteria such as *Blautia hansenii* and *Blautia producta* are significantly negatively correlated with visceral fat accumulation (39), and the increase in perirenal fat is considered one of the risk factors for chronic kidney disease (CKD) (40). *Intestinimonas butyriciproducens*, which was significantly decreased in both disease groups, is a key participant in the metabolism of fructose and lysine in the gut (41). At the functional level, we found that phenylalanine metabolism was enriched in the IgAN group. Studies have shown that the kidneys play an important role in converting phenylalanine to tyrosine. However, in patients with chronic kidney failure, this metabolic process may be impaired, leading to reduced tyrosine production, which in turn affects protein synthesis and other metabolic functions (42). Kim et al. proposed that chronic inflammation mediated by the gut–kidney axis may be a key mechanism underlying the progression from acute kidney injury (AKI) to chronic kidney disease (CKD) in the elderly (43). Li et al. reviewed the role of the gut microbiota in renal fibrosis, inflammation and oxidative stress, explored the potential of using microbiota-targeted interventions, such as probiotics, for CKD treatment (5). Zhang et al. further validated the therapeutic effect of *Bifidobacterium bifidum* tetragonum tablets in patients with diabetic kidney disease (DKD), demonstrating that the intervention improved clinical symptoms by suppressing inflammation and modulating the composition of the gut microbiota (44). In summary, these microbial changes may play a significant role in the pathogenesis of IgAN and MN by influencing SCFA metabolism, immune regulation, and inflammatory status.

### 4.2 Metabolic perturbations and diagnostic biomarkers

Currently, increasing attention has been given to the role of endogenous metabolites in kidney diseases. Cao et al. found that the metabolite 1-methoxypyrene (MP) can promote tubulointerstitial fibrosis (TIF) by activating the aryl hydrocarbon receptor (AhR) signaling pathway (45). Our metabolomic feature analysis revealed a significant upregulation of various dipeptides in the IgAN group, including Prolylleucine, Phe-Phe, Thr-Leu, Ala-Ile, Ala-Val, Alanyltrosine, L-Leucyl-L-Alanine and Tyrosylalanine. Similarly, many dipeptides were also upregulated in the MN group, such as Prolylleucine, Phe-Phe, Carnosine, Thr-Leu and Ala-Val. Several studies have shown that dipeptides are enriched in the fecal metabolites of patients with intestinal diseases, suggesting that dipeptide metabolic dysregulation may be a common feature of gut microbiome disturbance. Research by Mills RH et al. found that in patients with ulcerative colitis (UC), the symbiotic bacterium *Bacteroides vulgatus* can enhance protease activity, promoting protein hydrolysis and leading to the abnormal accumulation of dipeptides in fecal metabolites (46). Schirmer M et al. also observed the enrichment of dipeptides in the fecal metabolites of children with moderate to severe, newly diagnosed UC. This phenomenon may be mediated by the inhibition of proton-dependent oligopeptide transporter (POT) function due to the acidic environment in the colon (47), leading to disturbances in dipeptide metabolism between the host and microbiota (48). Bammens B provided evidence of impaired protein anabolism in chronic renal failure (CRF). This impairment may lead to protein malnutrition in CRF patients (49). Therefore, the dipeptide metabolic abnormalities observed in the fecal samples of our IgAN and MN patients may be associated with various factors, including changes in the gut microbiota and impaired protein anabolism. Additionally, numerous studies have explored the connection between amino acid metabolism abnormalities and kidney disease. Liu Y et al. found that the intestinal amino acid metabolic profile becomes dysregulated as CKD progresses in 5/6 Nx rats, suggesting that modulation of intestinal amino acid metabolism pathways could be a potential approach to intervene in the progression of CKD (50). Miao et al. found that Lactobacillus species improve MN by inhibiting the aryl hydrocarbon receptor pathway through tryptophan-derived indole metabolites (51). Moreover, previous studies have reported increased levels of free amino acids (FAAs) in the serum (52), plasma (53) and fecal metabolites (54) of IgAN patients. However, in our fecal samples from IgAN and MN patients, most FAAs did not show significant differences.

Some traditional biomarkers for predicting CKD are easily affected by external factors, limiting diagnostic accuracy. Therefore, researchers are focusing on identifying more reliable novel biomarkers. For example, Chen et al. identified five serum metabolites—5-MTP, canavaninosuccinate, acetylcarnitine, tiglylcarnitine and taurine—that can effectively distinguish CKD patients at various stages from healthy individuals (55). We also identified several potential biomarkers: 14 metabolites effectively distinguished IgAN from healthy controls, 3 metabolites effectively distinguished MN from healthy controls, and 4-Methyl-2-Oxopentanoic Acid differentiated IgAN from MN. The AUC values for these univariate biomarkers ranged from 0.8 to 0.85. Further analysis revealed that multivariate combinations of biomarkers exhibited superior diagnostic performance: the combination of 1,4-Dihydro-1-Methyl-4-Oxo-3-Pyridinecarboxamide and alpha-Linolenoyl ethanolamide distinguished IgAN from healthy controls (AUC = 0.919), the combination of 5-Methyl-2′-deoxycytidine and Prostaglandin D3 distinguished MN from healthy controls (AUC = 0.897), and the combination of 2-Methoxybenzaldehyde, acetoacetate, and Xanthine distinguished the two kidney diseases (AUC = 0.912). The results indicate that, compared to individual metabolites, the combination of biomarkers significantly enhances diagnostic performance, demonstrating high sensitivity (≥94%) and specificity (≥75%). This highlights the potential application value of multidimensional metabolomic analysis in disease diagnosis.

### 4.3 Microbiota-metabolite interactions in the gut-kidney Axis

The interaction between microorganisms and metabolites is widespread in the human body (56). This bidirectional regulatory mechanism is closely related to host physiological processes and plays a crucial role in the onset and progression of various chronic diseases (58–59). Currently, studies using db/db mice have provided evidence for the gut-metabolism-kidney axis (60). In addition, Zhi W et al. conducted clinical trials using features such as gut microbiota and metabolomics for monitoring, and preliminarily validated the safety and efficacy of fecal microbiota transplantation (FMT) in patients with IgAN (61). Shi et al. also investigated alterations in gut microbiota and metabolites in patients with idiopathic membranous nephropathy (IMN), and further developed a biomarker prediction model based on microbial features (29). Li et al. compared the gut microbiota and metabolite changes in patients undergoing Continuous Ambulatory Peritoneal Dialysis (CAPD), suggesting that the gut microbiome may serve as a potential target for the diagnosis and treatment of CAPD (62).

Our study shows that the levels of 4-Methylvaleric Acid and 2-Methylbutyric Acid in the fecal metabolites of the IgAN group are significantly lower than those in healthy controls, while the level of 2-Methylbutyric Acid in the MN group is significantly lower than that in healthy controls. SCFA are primarily produced through the fermentation of undigested dietary fibers by gut microbiota (63). These SCFAs serve as an important energy source for intestinal epithelial cells, regulating their proliferation, differentiation, and function, which in turn affects intestinal motility and enhances gut barrier function (65, 64). Among them, butyrate not only maintains gut health through its anti-inflammatory effects (66–67) but also influences regulatory T (Treg) cells, which are closely associated with the pathogenesis of IgAN (69). De Angelis M et al. found that the total levels of short-chain fatty acids in the feces of both progressive and non-progressive IgAN patients were significantly higher compared to those in healthy controls (54). Existing studies have shown that probiotics and their metabolic product SCFA can alleviate the clinical and pathological manifestations of IgAN by inhibiting the NLRP3/ASC/Caspase 1 signaling pathway (70). In addition, we also found that 4-Methylvaleric Acid and 2-Methylbutyric Acid in the IgAN group were positively correlated with microbes such as *Oscillibacter hominis, Intestinimonas butyriciproducens* and *Vescimonas coprocola*. In the MN group, 2-Methylbutyric Acid was positively correlated with *Oscillibacter hominis, Intestinimonas butyriciproducens, Vescimonas coprocola, Vescimonas fastidiosa* and *Dysosmobacter welbionis.*

Some species of Oscillibacter have been found to produce SCFA (71, 72). Existing studies have demonstrated that the main end product of *Vescimonas coprocola* is butyrate (73). *Intestinimonas butyriciproducens* primarily produces butyrate and acetate (74). The main end products of *Vescimonas fastidiosa* are acetate, n-butyrate, and isovalerate (75). *Dysosmobacter welbionis* primarily produces butyrate and has been shown to beneficially impact host metabolism (76). Therefore, we hypothesize that the reduced abundance of microbial populations such as Oscillibacter hominis, *Intestinimonas butyriciproducens* and *Vescimonas coprocola* in the gut of IgAN and MN patients may affect the levels of SCFA and participate in host metabolic regulation. In MN patients, the decrease in microbes such as *Vescimonas fastidiosa* and *Dysosmobacter welbionis* in the gut may also be one of the factors contributing to the changes in SCFA levels.

### 4.4 Limitations

Although this study provides new insights into the gut microbiota and metabolomic profiles of IgAN and MN, several limitations should be acknowledged. First, the sample size is relatively small (24 IgAN, 20 MN, and 17 healthy controls), which may limit the statistical power to detect subtle microbial or metabolic differences. Second, the study is cross-sectional, making it impossible to infer whether the observed dysbiosis and metabolic dysregulation are causes or consequences of renal pathology. Third, relying solely on fecal samples limits the understanding of host-microbiota interactions, as blood or urine metabolomic analyses could provide complementary information. Fourth, metabolite biomarkers need to be further validated in larger-scale studies. Future prospective research should also assess the prognostic value of these biomarkers and verify whether they outperform existing clinical indicators, thereby facilitating their clinical translation. Finally, although metagenomics offers higher resolution than 16S rRNA sequencing, functional validation is required to confirm the pathogenic role of specific taxa (e.g., Oscillibacter hominis) in the depletion of SCFAs.

## 5 Conclusion

This study analyzed the fecal metagenomic and metabolomic characteristics of IgAN and MN patients compared to healthy controls. Both kidney disease groups exhibited distinct gut microbiota composition patterns. Metabolomic analysis revealed significant enrichment of dipeptide metabolites in the feces of IgAN and MN patients, with some SCFA showing decreased levels, which were associated with the reduced abundance of microorganisms such as *Oscillibacter hominis, Intestinimonas butyriciproducens* and *Vescimonas coprocola*. Furthermore, we constructed a Logistic regression model to assess the potential application of metabolites for non-invasive diagnosis and differential diagnosis of IgAN and MN, identifying multiple potential biomarkers. The combination of metabolites significantly improved the diagnostic performance of the model, demonstrating high sensitivity and specificity.

## Data Availability

The raw metagenomic sequencing data have been deposited in the National Genomics Data Center (NGDC) (https://ngdc.cncb.ac.cn/) under accession number CRA025153.

## Footnotes

^1^ https://www.mzcloud.org/

^2^ https://www.genome.jp/kegg/pathway.html

^3^ https://hmdb.ca/metabolites

^4^ http://www.lipidmaps.org/

^5^ https://www.metaboanalyst.ca
